# Educating health professionals to optimise falls screening in hospitals: protocol for a mixed methods study

**DOI:** 10.1186/s12913-020-4899-y

**Published:** 2020-01-22

**Authors:** L. Shaw, D. Kiegaldie, M. E. Morris

**Affiliations:** 1 Faculty of Health Science, Youth and Community Studies, Holmesglen Institute, 488 South Road, Moorabbin, Vic 3189 Australia; 2 School of Allied Health, La Trobe Centre for Sport and Exercises Medicine Research, La Trobe University, Melbourne, Victoria 3086 Australia; 3 Healthscope, North Eastern Rehabilitation Centre, Ivanhoe, Victoria 3086 Australia

**Keywords:** Health professionals, Falls, Education, Evidence-based, Falls prevention, Hospital, Nursing, Physiotherapy

## Abstract

**Background:**

Falls in hospitals remain a major challenge to patient safety. All hospitalised adults are at risk of falling during their inpatient stay, though this risk is not always realised by patients and clinicians. This study will evaluate the outcomes of a hospital clinician education program that teaches clinicians how to screen for falls risk and assign mitigation strategies using clinical reasoning, rather than relying on a standardised falls risk assessment tool (FRAT). The education program aims to increase clinician knowledge, motivation and confidence in screening falls risk and selecting individual falls prevention interventions. Perceptions of the education intervention will also be examined.

**Methods:**

Participants will be a sample of convenience of nurses and allied health professionals from five Australian hospitals. For each hospital there will be two cohorts. Cohort 1 will be clinical leaders who shall receive a three-hour education program on the latest evidence in hospital falls risk assessment and how to implement a new falls screening and management tool. They will also be taught practical skills to enable them to deliver an effective one-hour in-service training session to Cohort 2. Cohort 2 will be recruited from the workforce as a whole and include nurses and other health professionals involved in routine hospital falls screening and prevention.

The investigation will be framed on Keller’s Model of Motivational Design and Kirkpatrick’s evaluation framework. It will involve a mixed methods pre and post-test questionnaire design inclusive of semi-structured telephone interviews, to triangulate the data from multiple approaches.

**Discussion:**

This study will quantify the outcomes of a high-quality clinician education program to increase knowledge of evidence-based practice for falls prevention. It is predicted that positive behavioural changes will occur in health professionals, leading to organisational change and improved patient outcomes. Furthermore, the findings from the study will inform the future refinement of educational delivery to health professionals across hospital sites.

**Trial registration:**

The study has also been approved by the Australian New Zealand Clinical Trials Registry: Preventing Hospital Falls: Optimal Screening UTN U1111-1225-8450.

**Universal Trial Number (UTN):** U1111–1228-0041 (obtained 5/2/19).

**Australian New Zealand Clinical Trials Registry (ANZCTR):** ACTRN12619000200189 (obtained 12/2/19).

## Background

Falls remain a serious problem in private and public hospitals world-wide and are associated with marked morbidity, mortality, increased length of stay and re-admissions [1–5]. Falls also incur substantial costs to hospitals and healthcare providers, insurers and individuals [6–8]. Whilst international estimates on falls are hard to find due to differences in reporting between countries, in the United Kingdom, the Royal College of Physicians’ National Audit of Inpatient Falls reported an average of 6.63 falls per 1000 occupied bed days [9].

This study is directed towards evaluating a clinician education program on how to screen hospital falls risk and assign mitigation strategies using clinical reasoning, rather than relying on a standardised Falls Risk Assessment Tool (FRAT). Historically, FRATs were used to try and identify patients at risk of falling whilst in hospital [10, 11]. As well as having poor predictive validity, it is now recognised that all hospitalised adults are at risk of falling during an episode of care [12]. The National Institute for Health and Care Excellence (NICE) guidelines state that, ‘fall risk prediction tools should not be used to predict inpatients’ risk of falling in hospital’ [13]. Furthermore, they advise that, ‘all patients aged 65 years or older and patients aged 50 to 64 years who are judged by a clinician to be at higher risk of falling due to an underlying condition, should be judged as being at risk of falling and their care managed according to recommendations' [13].

Research into the impact of education interventions on falls within hospitals has mainly focussed on patient education [14] and many interventions fail to adequately describe the educational methods employed [15]. As a precursor to this study, a scoping review was performed to determine the extent of the research evidence and design elements, for education interventions for health professionals in falls screening and prevention. There were few studies on this topic. One study described the outcomes of a half day education programme about fall and fracture prevention for staff given by specialist osteoporosis nurses in care facilities [16]. It did not find evidence for a reduction in the rate of falls [16] and the quality of evidence was assessed in a recent Cochrane review as being very low [17]. A pilot cluster-randomised trial in residential aged care evaluated an educational programme to improve staff connections, communication, and problem solving for the implementation of a falls quality improvement programme [18]. This trial of 546 eligible clinicians in four intervention nursing homes, did not find a change in falls rates. Notably, few education studies discussed in depth the educational approaches used, or whether theoretical principles were incorporated into their design [19].

The aims of the current study are to: (i) Investigate the self-reported views of hospital clinicians of their knowledge, clinical practice, confidence, motivation and attitudes towards screening for falls risk using traditional and contemporary approaches; (ii) Examine perceptions of an education intervention designed to communicate the latest evidence on falls screening and how to implement a new evidence-based Falls Screening Tool into daily clinical practice; (iii) Determine the effectiveness of the educational program content and delivery in supporting behaviour change for falls screening in hospitals. We shall also explore the views and experiences of health professionals on hospital falls risk screening and historical FRATs more generally.

## Methods/ design

### Design

This study is part of a large National Health and Medical Research Council of Australia partnership grant program of work, on falls prevention in hospitals (Morris et al., GNT1152853). For the larger trial, ten Australian hospitals will be randomised to an intervention group (five hospitals using a new Falls Screening Tool), or a control group (five hospitals continuing to use the historical FRAT form) by another organisation (The University of Melbourne). The new Falls Screening Tool removes the risk assessment component from the historical FRAT form and associated summary scores, yet maintains other components for falls mitigation. This education trial will be conducted at the 5 intervention hospitals.

### Participants and recruitment methods

At each of the intervention hospitals, there will be two separate cohorts. The names and contact details of all potential participants will be obtained from an existing hospital database.

*Cohort 1*: (*n* = 10 clinical leaders at each experimental hospital), will be approached by the Hospital General Manager to consider participating in the study. These clinical leaders will be invited to participate via email, which will contain all details about the study, the requirements for participation, and the Participant Information and Consent Form (PICF) (Additional file 1). On the day of the education, cohort 1 will be required to complete the written PICF, Pre-test and Post-test 1 surveys and return them to the researcher who is leading the education program. The PICF will also include consent to participate in a follow up telephone interview. Only those providing their contact details and consent will be contacted to participate in the interviews. Completing the Post-test 2 survey online will imply consent.

*Cohort 2:* (*n =* All nurses and allied health professionals involved in completing falls screening), will be eligible to participate and will be invited to attend a one-hour in-service education program. Staff will be asked to read the PICF (Additional file 2), which outlines the full details of the research project and the requirements for participation. Participants will be drawn from all wards in the five intervention hospitals, excluding paediatric, maternity, emergency and theatre wards. We are aiming for 65% attendance of all nurses and allied health professionals at each experimental site. On the day of the in-service training, cohort 2 will be required to complete the written PICF, Pre-test and Post-test 1 surveys. These will then be returned to the researchers via registered post. The PICF will also include consent to participate in a follow up telephone interview. Only those providing their contact details and consent will be contacted to participate in the interviews. Completing the Post-test 2 survey online will imply consent.

#### Intervention

The theoretical concepts and principles underpinning the educational intervention design and research methods of the study are based on behavioural and social sciences theory. Keller’s motivational design for learning and performance will be incorporated into the instructional design and research measurement outcomes. For the education protocol, participants will receive a high-quality education program on evidence-based hospital falls screening underpinned by quality education design principles put forward by Kiegaldie and Farlie [15]. The education intervention aims to increase participant knowledge, motivation and confidence about evidence-based practice on the use of a new falls screening tool that focusses on clinical reasoning and patient-centred, personalised falls prevention programs. The educational program also aims to support effective implementation of the new learnings into clinical practice. In order to implement this new intervention, a multifaceted educational approach will be employed that utilises a mixture of interactive teaching methods suited to the learning needs of busy clinicians [20].

The new Falls Screening Tool aims to enable clinicians to screen hospital patients, and assign appropriate falls mitigation interventions. The screening items identify, for example, whether or not the person is hospitalised, those aged 65 years or older, those who have had a fall in the past 12 months, anyone with sight, hearing or sensory deficits, and anyone who has received an anaesthetic in the last 24 h. The screening tool provides an opportunity for clinicians to use their judgement to determine the appropriate falls prevention interventions that are listed in a separate section on the form.

### Education intervention

#### Cohort 1

Will receive a three-hour education program using best educational design [15], which will educate clinical leaders on the latest evidence on hospital falls risk assessment and guide them in how to implement a new Falls Screening Tool. Teaching methods will include: content delivery on the latest evidence for falls screening; interactive face-to-face teaching; small group critical thinking activities on the challenges of falls prevention in hospitals, and the arguments for and against the use of FRATs; practical exercises using clinical vignettes to compare and contrast the historical FRAT and the new Falls Screening Too; and feedback gauging their views on each of the forms. Participants in cohort 1 will also be provided with the practical skills and associated educational resources, to enable them to deliver an effective, one-hour in-service training session on these topics, to cohort 2.

#### Cohort 2

**Fig. 1:**
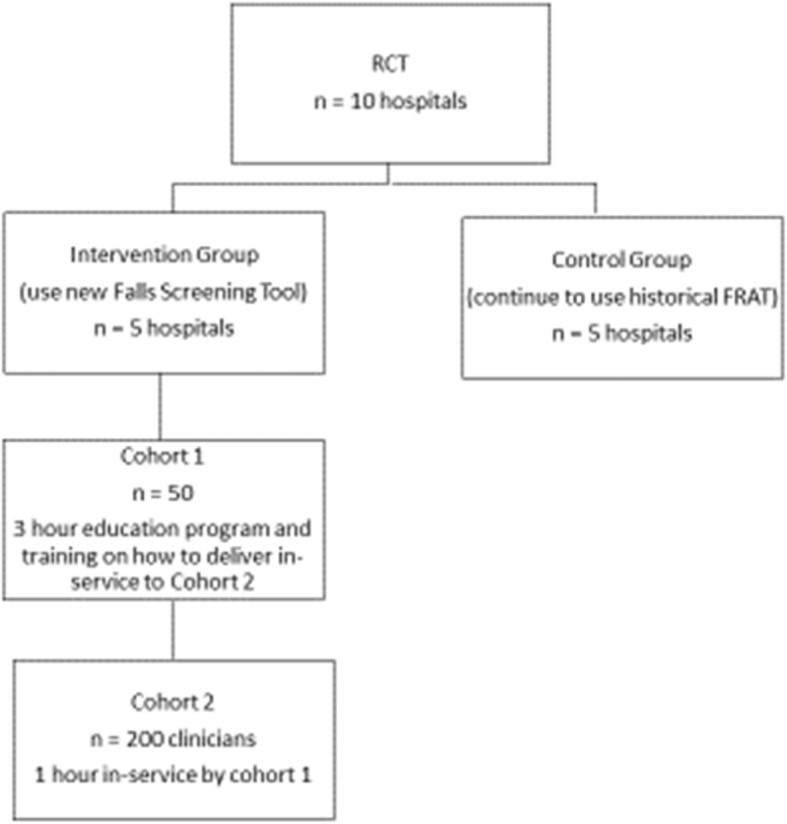
Study protocol for the Parent Trial. This sub study pertains to clinician education used for the intervention group

Will attend a one-hour in-service training and be educated by cohort 1. Methods of educational delivery will also include: interactive face-to face teaching; content delivery on the latest evidence for falls risk assessment; small group critical thinking activities; and practical exercises using clinical vignettes (Fig. 1).

### Data collection

A Pre and Post-test design will be used, elaborated through a mixed methods research approach, with the qualitative data helping to build on the initial quantitative results [21]. Data will be collected via de-identified surveys (using the participants’ initials and last three digits of their mobile telephone number) and individual telephone interviews. Multiple time points will be used for data collection from surveys:

(1) Pre-test: Immediately prior to the education program.

(2) Post-test 1: Immediately post the education program.

(3) Post-test 2: 2 months post implementation of the new screening tool.

For cohort 1, paper-based surveys will be collected by those who deliver the education program. For cohort 2, all surveys will be returned to researchers via registered post.

### Instruments

The ***Pre-test Survey*** identifies demographic characteristics of the sample such as profession, position, years of clinical practice experience, gender and location of work. It asks participants to select a single statement from a list of 5 to identify their view on the historical FRAT form and their view on what their colleagues might think. They are then asked to rate how strongly they agree or disagree on 20 statements regarding evidence-based practice falls risk assessment on a 5-point Likert scale from 1 (strongly disagree) to 5 (strongly agree). Free text comments are invited at the conclusion of the survey.

The ***Post-test 1 Survey*** repeats the 5-item statement to identify participants’ views on the forms. It also repeats 13 items from the pre-test survey on evidence-based practice and falls risk assessment with minor changes to 6 items and the addition of 4 new items (1 item is removed). Participants are asked to identify and comment on whether the new Falls Screening Tool will be beneficial to patients and to them personally. ***Post-test 1 Survey*** also includes 9 items from a previously validated Instructional Material Motivation Survey (IMMS) [22]. The relevance subscale from this survey is the only section to be used, as it has the most applicability and is an approach used in a previous thesis on falls prevention [23]. This section also includes 5 items seeking participants’ perceptions of the overall learning experience. Four open ended questions are included asking participants to comment on the effectiveness of the program such as what worked well, what needs improvement and their views on the ‘take home’ messages.

For Cohort 1, post-test survey 1 also asks participants additional questions around how prepared and confident they feel in educating others (Cohort 2) on this topic.

***Post-test 2 Survey*** repeats all items from Post-test 1 and makes minor changes to 3 items in the evidence-based practice and falls risk assessment section. Three open-ended questions are asked about the implementation of the new form along with whether the new form has been beneficial to patients and to them personally.

***Semi-structured telephone interviews*** (Additional file 3) will be conducted post implementation with consenting participants randomly selected from two randomly chosen experimental sites, in order to triangulate the data from the questionnaires and allow staff to state confidentially their thoughts on the changes to falls screening. Sequential explanatory design will be employed, where the qualitative data from the telephone interviews will help to explain or build on the initial quantitative results [24]. The quantitative phase will occur first using the surveys, and the qualitative component occurring subsequently via semi-structured telephone interviews, to gather detailed information about the education sessions and the new falls screening tools.

From Cohort 1, one health professional will be invited to participate. Cohort 2 interviewees invited to participate will consist of two other randomly selected staff - a junior staff member (qualified 3 years or less), and a senior staff member (qualified more than 3 years). Telephone Interviews will be audio-recorded for transcription purposes, and to ensure clarity and accuracy. See Table 1: Study Protocol.

**Table 1:** Study protocol

Cohort 1	Cohort 2
Pre-test Characteristics of participants Views on historical FRAT form (*n* = 2 items) Views on EBP of falls (*n* = 20 items) Free text comments on falls screening and assessment	Pre-test Characteristics of participants Views on historical FRAT form (*n* = 2 items) Views on EBP of falls (*n* = 20 items) Free text comments on falls screening and assessment
3 h education program	1 h in-service
Post-test 1 Repeated items a/a (*n* = 13) Changed items (*n* = 6) Additional items (*n* = 4) 1 item removed IMMS Free text comments on falls assessment and the learning experience	Post-test 1 Repeated items a/a (*n* = 13) Changed items (*n* = 6) Additional items (*n* = 4) 1 item removed IMMS Free text comments on falls assessment and the learning experience
Post-test 2 Repeated items a/a (*n* = 13) Changed items (*n* = 6) Additional items (*n* = 4) 1 item removed IMMS Free text comments on falls assessment and the learning experience	Post-test 2 Repeated items a/a (*n* = 13) Changed items (*n* = 6) Additional items (*n* = 4) 1 item removed IMMS Free text comments on falls assessment and the learning experience
Individual Interviews	Individual interviews

### Outcomes

#### Primary outcome measures

i. Conceptual and behavioural change from utilising the historical FRAT.
ii. Change in knowledge of evidence-based practice for falls screening and prevention in hospitals
iii. Provide clinical leaders in cohort 2 with the skills to enable them to deliver an effective education program to other clinicians.

#### Secondary outcome

Participants’ evaluations of the education program to inform the future refinement of educational delivery to clinicians.

### Data analysis

*Quantitative data:* Using SPSS, demographic data and responses on all Likert scaled surveys and rating scales, will be analysed descriptively. To see if there is a significant difference between mean scores at Pre-test, Post-test 1 and Post-test 2, where there are three or more mean values to compare together, a one way Analysis of Variance (ANOVA) will be used. Where there are only two mean values to compare, an Independent Samples t-test will be used. Comparisons between groups will be measured according to profession, years of clinical practice, and hospital site. We will determine whether the education intervention resulted in a statistically significant conceptual change to the new form, and whether the education intervention requires further development in terms of content and delivery.

With respect to statistical power and sample size calculations: we are seeking significant differences at the 95% confidence level, so *α* = 0.05. Further, we aim to achieve a statistical power of *π* = 0.8, and to detect effect sizes of Cohen’s *d* = 0.4 and larger. The corresponding sample size required is calculated to be *n* = 50 (using the program *Power and Sample Size v.3.0, 2009*) [25]. Cohort 1 will have the requisite *n* = 50 and Cohort 2 is expected to be at least twice as large as this.

*Qualitative data:* Thematic analysis will be employed to analyse the qualitative data from the semi-structured telephone interviews and the textual responses to open ended questions in the surveys. Themes reflect recurrent and distinctive features of participants’ accounts, characterising particular perceptions and/ or experiences seen as relevant to the research questions [26].

The audio-recorded interviews will be transcribed verbatim in Word and the text transferred into Excel. One researcher will develop initial descriptive codes of the responses to each prompt of the semi-structured interview. A second researcher will check the transcripts, review the initial descriptive codes, and the two researchers will discuss and finalise the framework for analysis. The responses from the focus group participants will be coded using this revised framework and categories identified. Finally, a frequency count for each focussed code will be conducted.

### Risk management and safety

It is anticipated that there will be no physical, psychological, social, legal or financial harm to the participants involved in this study. Participants may withdraw from the study at any time.

However, with any study there are risks. We have listed the risks we know about below.

- There is a low risk that not using the current FRAT will unexpectedly increase falls in hospitals.
- There is a low risk that clinicians could become anxious about using new methods of recording.

To mitigate risk, an independent safety monitoring committee will regularly check the falls rates in each hospital and compare rates with historical values, to ensure falls or associated injuries have not systematically increased as a result of the trial. Ward-level falls rates will also be used for safety monitoring (monthly reports) provided to the safety monitoring committee.

### Data security and handling

Survey data will be directly recorded into SPSS. Interview notes and audio will record the telephone interviews, which will be transcribed. All information collected will be anonymous and no individual will be identifiable in any of the reporting of outcomes. During the study all files will be kept secure for the duration of the project. Following completion of the study, project documentation will be kept in a secure, lockable location in the office of one of the lead researchers. Data will be stored for 7 years. No data will be used for other projects. All electronic data will be kept in password protected databases, separate from any identifying information. Access to data will be limited to the chief investigators and support staff only.

## Discussion

To date there has been little research on the design and outcomes of clinician education programs to screen and mitigate hospital falls. Falls education without a theoretical foundation may bring into question the scientific quality of that intervention. To improve patient outcomes, the education of staff needs to result in behavioural change, such as the transfer of knowledge and skills gained from training into practice [27, 28]. This study will determine the impact of an evidence-based tailored intervention and best educational design, for the implementation of a falls prevention education program to health professionals in a hospital environment. It underscores the important role that education has in educating staff on a clinical intervention to support the research process. The findings will be disseminated in peer reviewed journals, throughout hospitals, and via professional and scientific conferences.

We propose that this study is an innovative way to target the learning environment and maximise clinicians’ ability to adopt the content learned in the education program and integrate it into knowledge and action [29]. Additionally, the education methods used can be applied in future projects to implement evidence based practices for other clinical problems.

### Strengths and limitations

This study is unique and the educational program can be delivered in a busy, time pressured clinical environment using clinical champions (leaders) and a ‘train the trainer’ approach. We shall clearly report the educational features so that others can adopt a best practice approach to educational design. The education interventions are designed to be readily implemented across other hospital sites and applied to other clinical education interventions. This study is not without limitations. There is a time factor related to educating Cohort 2, and education on a new clinical form may not be seen as a high priority for clinicians in a busy clinical environment with competing demands on time. There is also the potential for inaccurate self-reporting caused by recall bias [30] influenced by different clinicians’ perceptions. Loss to follow up is also likely, particularly for post survey 2 when moving from paper based surveys to online surveys.

## Supplementary information

**Additional file 1.** C1_PICF. Cohort 1 Participant Information Statement and Consent Form

**Additional file 2.** C2_PICF. Cohort 2 Participant Information Statement and Consent Form

**Additional file 3.** Semi-structured interview questions. Semi-structured interview questions

## Data Availability

Not applicable

## Abbreviations

FRAT: Falls Risk Assessment Tool
PICF: Participant Information and Consent Form
