# Supplementary material for: Educating health professionals to optimise falls screening in hospitals: protocol for a mixed methods study
Source: BMC Health Serv Res. 2020 Jan 22;20:54. doi: 10.1186/s12913-020-4899-y (PMC6977326; doi:10.1186/s12913-020-4899-y)
Supplement: Supplementary file 1 — Additional file 1. C1_PICF. Cohort 1 Participant Information Statement and Consent Form [file 12913_2020_4899_MOESM1_ESM.docx]

| **Educating health professionals to optimise falls screening** **HEC Number: HEC18501**  The research is being carried out by the following researchers: | | |
| --- | --- | --- |
| **Role** | **Name** | **Organisation** |
| Investigator | Professor Meg Morris  Associate Professor Debra Kiegaldie  Dr Louise Shaw | La Trobe University, Australia  Holmesglen Institute, Australia  La Trobe University, Australia |
| **Research funder** | This research is supported by funding from the National Health and Medical Research Council of Australia (GNT1152853), Healthscope, Holmesglen Institute and La Trobe University. | |

1. **What is the study about?**

You are invited to participate in a study which explores your experiences of using an existing Falls Risk Assessment Tool (FRAT) and evaluates your views of an education program designed to prepare you for using a new Falls screening form.

Falls are one of the most serious problems in Australia’s private and public hospitals and are associated with marked morbidity, mortality and increased length of stay. Falls also incur substantial costs to hospitals and healthcare providers, insurers and individuals. The aims of the study are to investigate your views of falls risk assessment, the existing Falls Risk Assessment Form, and a new falls screening tool. It will also examine your opinions of an education program designed to teach the latest evidence on falls risk assessment and how to implement the new falls form into daily practice.

Your contact details were obtained from Ms Cathy Jones, the National Manager Quality & Compliance, Healthscope.

1. **Do I have to participate?**

Being part of this study is voluntary. If you want to be part of the study we ask that you read the information below carefully and ask us any questions.

You can read the information below and decide at the end if you do not want to participate. If you decide not to participate this won’t affect your relationship with Healthscope, Holmesglen Institute, La Trobe University or any other listed organisation.

1. **Who is being asked to participate?**

You have been asked to participate because you are a clinical leader at a participating Healthscope Hospital and are involved in completing falls risk assessment forms.

1. **What will I be asked to do?**

If you wish to take part in this study, we ask you to attend a three hour education program. This will involve educating you on the latest evidence on hospital falls risk assessment and guide you on how to implement a new falls screening form. You will also be provided with the practical skills to enable you to deliver an effective, one-hour in-service training session to nurses and allied health professionals at your hospital, who are involved in completing the new falls screening tool. You will be taught the education program methods and given a standard lesson plan with all associated educational resources.

You will be required to complete 3 surveys over 3 time points:

1. Before undertaking the education program
2. Immediately after the education program
3. Approximately 2-3 months after undertaking the education program.

If randomly selected, we also invite you to participate in an optional individual telephone interview. The telephone interview will further explore your views on the new form and the education program.

1. **What are the benefits?**

The benefits of you taking part in this study is that it will give you an opportunity to provide valuable information about falls risk assessment to improve your own clinical practice and patient care.

The expected benefits to society in general are an understanding of the benefits, consequences, barriers and facilitators to moving to a new falls screening tool. Your responses will assist in allowing us to measure:

- Behavioural change from the existing FRAT form to a new falls screening tool
- Changes in knowledge of evidence-based practice for falls screening and prevention in hospitals

Outcomes from the education program will also help to inform the future refinement of the delivery of education interventions across all Healthscope sites.

1. **What are the risks?**

With any study there are (1) risks we know about, (2) risks we don’t know about, and (3) risks we don’t expect. It is anticipated that there will be no harm to you as a participant involved in this study. If you experience something that you aren’t sure about, please contact us immediately so we can discuss the best way to manage your concerns.

| **Name/Organisation** | **Position** | **Telephone** | **Email** |
| --- | --- | --- | --- |
| Professor Meg Morris | Physiotherapist | 61 433405 662 | m.morris@latrobe.edu.au |

We have listed the risks we know about below. This will help you decide if you want to be part of the study.

- There is a low risk that not using the current Healthscope FRAT will unexpectedly increase falls in hospitals.
- There is a low risk that clinicians could become anxious about using new methods of recording.

To mitigate risk, an independent safety monitoring committee will regularly check the falls rates in each hospital and compare rates with historical values to ensure falls or associated injuries have not systematically increased as a result of the trial. Ward-level falls rates will also be used for safety monitoring (monthly reports) provided to the safety monitoring committee.

1. **What will happen to information about me?**

By completing this from, this tells us that you want to take part in the study. We wil collect and store information in ways that will not reveal who you are. This means you cannot be identified in any type of publication from this study. We will keep your information for 7 years after the project is completed. After this time we will destroy all of your data.

We will collect, store and destroy your data in accordance with La Trobe University’s Research Data Management Policy which can be viewed online using the following link: <https://policies.latrobe.edu.au/document/view.php?id=106/>. All electronic data will be kept in password protected databases, separate from any identifying information. Access to data will be limited to the chief investigators and support staff only.

The information you provide is personal information for the purposes of the Privacy and Date Protection Act 2014 (Vic). You have the right to access personal information held about you by the University, the right to request correction and amendment of it, and the right to make a complaint about a breach of the Information Protection Principles as contained in the Information Privacy Act.

1. **Will I hear about the results of the study?**

If you wish to know about the results of the study, you can email Professor meg Morris [m.morris@latrobe.edu.au](mailto:m.morris@latrobe.edu.au) in February 2019 and she will forward a de-identified summary report to you.

1. **What if I change my mind?**

At any time, you can choose to no longer be part of the study. You can let us know by:

1. Completing the ‘Withdrawal of Consent Form’ (provided at the end of this document) and emailing it to Professor Meg Morris: m.morris@latrobe.edu.au;
2. Calling Professor Meg Morris: 0433 405662;
3. Emailing Professor Meg Morris: m.morris@latrobe.edu.au

Your decision to withdraw at any point will **not** affect your relationship with La Trobe University or any other organisation listed.

1. **Who can I contact for questions or want more information?**

If you would like to speak to us, please use the contact details below:

| **Name/Organisation** | **Position** | **Telephone** | **Email** |
| --- | --- | --- | --- |
| Professor Meg Morris | Physiotherapist | 61 433405 662 | m.morris@latrobe.edu.au |

1. **What if I have a complaint?**

If you have a complaint about any part of this study, please contact:

| **Ethics Reference Number** | **Position** | **Telephone** | **Email** |
| --- | --- | --- | --- |
| [INSERT - Ethics Number] | Senior Research Ethics Officer | +61 3 9479 1443 | [humanethics@latrobe.edu.au](mailto:humanethics@latrobe.edu.au) |

**Consent Form – Declaration by Participant**

I (the participant) have read (or, where appropriate, have had read to me) and understood the participant information statement, and any questions have been answered to my satisfaction. I agree to participate in the study, I know I can withdraw at any time. I agree information provided by me or with my permission during the project may be included in a thesis, presentation and published in journals on the condition that I cannot be identified.

I would like my information collected for this research study to be:

Only used for this specific study;

Used for future related studies;

**Participant Signature**

I have received a signed copy of the Participant Information Statement and Consent Form to keep

| Participant’s printed name |  |
| --- | --- |
| Participant’s signature |  |
| Date |  |

**Consent to be contacted for telephone interview**

I consent to be contacted for a follow up telephone interview.

| Contact Phone No. |  |
| --- | --- |

**Declaration by Researcher**

I have given a verbal explanation of the study, what it involves, and the risks and I believe the participant has understood;

I am a person qualified to explain the study, the risks and answer questions

| Researcher’s printed name |  |
| --- | --- |
| Researcher’s signature |  |
| Date |  |

* All parties must sign and date their own signature

**Withdrawal of Consent**

I wish to withdraw my consent to participate in this study. I understand withdrawal will not affect my relationship with La Trobe University of any other organisation or professionals listed in the Participant Information Statement. I understand the researchers cannot withdraw my information once it has been analysed, and/or collected as part of a focus group.

**I understand my information will be withdrawn as outlined below:**

- Any identifiable information about me will be withdrawn from the study
- The researchers will withdraw my contact details so I cannot be contacted by them in the future studies unless I have given separate consent for my details to be kept in a participant registry.
- The researchers cannot withdraw my information once it has been analysed, and/or collected as part of a focus group

***if you have consented for your contact details to be included in a participant registry you will need to contact the registry staff directly to withdraw your details.*

I would like my already collected and unanalysed data

Destroyed and not used for any analysis

Used for analysis

**Participant Signature**

| Participant’s printed name |  |
| --- | --- |
| Participant’s signature |  |
| Date |  |

**Please forward this form to:**

| CI Name | [INSERT - CI Name] |
| --- | --- |
| Email | [INSERT - work email address] |
| Phone | [INSERT - work phone] |
| Postal Address | [INSERT - work postal address] |
