# Supplementary material for: Educating health professionals to optimise falls screening in hospitals: protocol for a mixed methods study
Source: BMC Health Serv Res. 2020 Jan 22;20:54. doi: 10.1186/s12913-020-4899-y (PMC6977326; doi:10.1186/s12913-020-4899-y)
Supplement: Supplementary file 3 — Additional file 3. Semi-structured interview questions. Semi-structured interview questions [file 12913_2020_4899_MOESM3_ESM.docx]

**SEMI-STRUCTURED INTERVIEW QUESTIONS**

**Educating clinicians to optimise falls risk screening**

**INTRODUCTION**

- Welcome and introductions
- Housekeeping and overview of the interview process
- Acknowledge consent
- Inform the participant that the interview is being audio-recorded

**QUESTIONS**

1. Do you think the NEW falls screening tool has benefited patients?
2. Do you think the NEW falls screening tool has been beneficial to you?
3. Did the education program alter your confidence in assessing and managing falls? E.gs/ prompts:
   1. Do you now feel more confident assessing patients at risk of falls?
   2. Do you feel prepared for preventing falls in your clinical area?
   3. Do you feel confident in judging the best strategies to implement for falls prevention?
   4. Do you feel confident implementing the findings from the new falls screening tool?
4. Has the implementation of the NEW falls screening tool been effective in your clinical area/ has it benefitted your work environment?
   1. Do you feel motivated to implement the form/ findings into your clinical area?
   2. Overall, what has been the response of your colleagues to the NEW falls screening tool?
5. Has your hospital supported you to implement the NEW falls screening tool? How?
6. Do you think the NEW falls screening tool is based on the best available research evidence?
7. What have been the enablers of implementing the NEW falls screening tool? (possible prompts -time taken, education session)
8. What have been the challenges of implementing the NEW falls screening tool?
9. Have you managed to overcome these? How?
10. Did the education program on the NEW falls screening tool assist in preparing you for a new process?
    1. What parts of the education session did you find most helpful?
    2. Do you have any suggestions for how the education could be improved?
11. Conclusion: Overall, do you think the NEW falls screening tool is better than the old FRAT? Why or why not?

**CLOSE**

- Thank the participant for their time
- Provide contact details of the lead researcher in case the participant has any questions
- Explain what will happen with results (transcription, de-identified, collated into broader themes)
- Provide an opportunity to clarify any content
